# Motion Planning-Augmented Hierarchical Reinforcement Learning for Long-Horizon Mobile Manipulation

**DOI:** 10.3390/s26123845

**Published:** 2026-06-17

**Authors:** Hyungtai Kim, Mun-Taek Choi

**Affiliations:** School of Mechanical Engineering, Sungkyunkwan University, Suwon 16419, Republic of Korea; gudxo1229@skku.edu

**Keywords:** mobile robots, reinforcement learning, motion planning, service robots, robot kinematics

## Abstract

Long-horizon mobile manipulation requires a robot to execute a sequence of heterogeneous subtasks such as navigation, picking, and articulated-object manipulation in indoor environments. Standard reinforcement learning suffers from reward sparsity and inefficient exploration in this setting, and hierarchical methods often fail at the hand-off between consecutive subtasks when the terminal state of one subtask is kinematically infeasible for the next. We propose a motion planning-augmented hierarchical reinforcement learning architecture to resolve the fundamental trade-offs between sample efficiency and hand-off reliability in long-horizon mobile manipulation. The mission is decomposed into subtasks via a Semi-Markov Decision Process; within each subtask, a collision-free reference trajectory generated by RRT* in the full joint configuration space is embedded into the reward as a per-step shaping signal; and a region-goal mechanism, defined analytically from inverse kinematics feasibility, replaces rigid coordinate hand-offs with a continuous feasible region. The architecture is evaluated in the ManiSkill-HAB simulation under teleport-free sequential execution and challenging initialization. The proposed method improves subtask success rate and sample efficiency over the baseline across all six evaluated subtasks, and the advantage compounds along the long-horizon task chain.

## 1. Introduction

Aging populations and labor shortages in many countries have raised the importance of service robots in domestic environments [1]. Long-horizon mobile manipulation has become a key capability for these robots, requiring the robot to navigate unstructured indoor layouts and perform complex object interactions across extended task sequences [2,3]. These multi-stage missions require both spatial understanding and continuous physical control. However, model-free reinforcement learning struggles with exploration and execution in such settings [4].

Standard RL approaches struggle with reward sparsity in long-horizon missions, since the probability of obtaining a meaningful learning signal decreases with the length of the required action sequence [5]. Hierarchical architectures address this by decomposing the mission into smaller subtasks. However, individual policies still face significant exploration difficulties in high-dimensional, cluttered configuration spaces [2,4].

Sequentially chaining isolated skills introduces another challenge known as the hand-off problem [6]. Conventional methods rely on rigid point-to-point navigation targets for hand-offs between subtasks, which can leave the mobile base in a kinematically infeasible pose for the next manipulation phase [2]. Such physical mismatches accumulate as the task chain grows longer [3]. Existing approaches often handle this by teleporting the robot to a predefined configuration between subtasks, which does not reflect the constraints of continuous physical execution [6].

We introduce a motion planning (MP)-augmented hierarchical reinforcement learning architecture and evaluate it via simulation on the ManiSkill-HAB benchmark [1]. The proposed system tackles the three challenges above through three contributions. First, a hierarchical SMDP-based decomposition chains modular subtasks to reduce long-horizon reward sparsity, enabling each policy to acquire foundational behaviors independently. Second, collision-free geometric trajectories generated by OMPL/RRT* are embedded directly into the RL reward function as dense per-step shaping signals, guiding the agent toward promising regions of the joint configuration space and accelerating policy convergence. Unlike prior reward-shaping works confined to fixed-base arms or 2D mobile robots in Cartesian space, the proposed formulation operates in the full joint configuration space $C_{\mathrm{free}}$ of the mobile manipulator, providing whole-body guidance without inverse kinematics ambiguity. Third, a region-goal mechanism replaces rigid coordinate-based subtask hand-offs with a continuous spatial manifold, providing kinematically feasible transitions between navigation and manipulation phases without non-physical teleportation.

## 2. Related Work

### 2.1. Hierarchical Reinforcement Learning for Long-Horizon Mobile Manipulation

Reinforcement learning has been applied to mobile manipulator control at the whole-body level, jointly optimizing base locomotion and arm motion through a single end-to-end policy [7]. While this demonstrates the feasibility of unified joint-space control via RL, such flat policy architectures struggle to scale to long-horizon tasks that require coordinated execution of multiple heterogeneous subtasks.

Hierarchical decomposition has therefore emerged as the dominant strategy for managing this complexity [8,9]. ReLMoGen [2] separates locomotion and manipulation into distinct modules but relies on artificial teleportation between subtasks. PLANRL [10] switches between classical navigation and RL-based contact control, yet the motion planner directly executes navigation actions, precluding a unified whole-body policy. Studies evaluated on the HAB benchmark [6] similarly bypass the physical connectivity between subtasks through teleportation oracles. These approaches share a common vulnerability: the terminal state of one subtask frequently falls outside the feasible initiation set of the next, causing compounding failures across the task chain [9]. The proposed architecture resolves this hand-off problem through a region-goal mechanism that guarantees kinematically feasible hand-offs without non-physical resets.

### 2.2. Integrating Motion Planning with Reinforcement Learning

Prior work on coupling motion planning with RL differs along two axes: the integration modality and the target platform. The integration modality refers to whether the planner executes actions, supplies sub-goals, or shapes the reward. Several action-space methods such as MoPA-RL [4] and PSL [11] allow the planner to directly execute a subset of actions for fixed-base arms, which precludes learning a unified whole-body policy. Sub-goal methods such as PRM-RL [5] and PRM+TD3 [12] supply roadmap waypoints as navigation targets to local RL policies for 2D point robots, without coupling path geometry to the reward signal. Reward-shaping methods are closer in spirit to the present work. Ota et al. [13] embed RRT*-generated waypoint deviations as per-step penalties for a fixed-base 6-DoF manipulator; HMP-DRL [14] integrates A* global paths as sparse checkpoint signals for 2D mobile navigation; Curriculum RL [15] applies staged path-tracking penalties to 2D navigation; and Song et al. [16] embed physics-inspired Coulomb force signals into the RL reward for 2D mobile robot navigation. More recently, Wang et al. [17] combine adaptive reward shaping with curriculum learning to balance positioning accuracy against dynamic obstacle avoidance for a fixed-base manipulator under partial observability. These reward-shaping works, however, remain confined to fixed-base arms or 2D platforms in Cartesian space, and none extends to the full joint configuration space of a mobile manipulator under long-horizon execution.

Residual RL [18] takes a structurally different approach, additively combining a fixed hand-engineered controller with a learned residual, but requires a pre-existing functional controller that is generally unavailable for long-horizon mobile manipulation in unstructured environments. In a similar vein, WHOLE-MoMa [19] uses a sub-optimal whole-body controller as a structural prior to collect task-relevant data and refines it with offline reinforcement learning for articulated-object mobile manipulation; like residual RL, it presupposes an available whole-body controller and does not couple a planner-generated path into the reward signal.

Table 1 compares the proposed method with representative prior RL-based work along eight axes. The first two axes, the simulator and the task family, define the evaluation environment and form the basis for a fair direct comparison: among RL-based methods, only MS-HAB [1] and the present work are evaluated on the same simulator (ManiSkill3) and the same HAB task family. The remaining axes characterize the three primary contributions of this work (MP integration modality, reference space, region-goal navigation), the policy architecture (HRL), the skill-chaining protocol with sequential navigation (SC), and the grasp model. The combination of reward-level integration with the full joint configuration space is shared only with Ota et al. [13], whose method targets a fixed-base manipulator in a different simulator and task. Adding the region-goal hand-off mechanism narrows the comparable set to M3 [20], which similarly addresses subtask chaining through region-goal navigation in a mobile-manipulator setting but does not integrate the motion planner into the reward signal, does not operate in the full joint configuration space, and was evaluated under the original HAB protocol [6] with magical grasping. Among RL-based methods evaluated on the same ManiSkill3 simulator and HAB task family, the proposed architecture is the first to integrate reward-shaping in the full joint configuration space of a mobile manipulator, couple it with a region-goal that analytically delineates the operational region of the subsequent manipulation subtask, and evaluate the combined system under realistic-grasp, teleport-free long-horizon execution.

## 3. Preliminaries

### 3.1. Markov Decision Process

The control problem is modeled as a Markov Decision Process (MDP) [21], defined by the tuple M=(S,A,T,R,γ): S is the continuous state space, A is the continuous action space, $T(s_{t+1}|s_{t},a_{t})$ is the transition function, $R(s_{t},a_{t})$ is the reward, and γ∈[0,1) is the discount factor. The policy $\pi (a_{t}|s_{t})$ is trained to maximize the expected discounted return J(π):

(1) $$J\left(\pi \right)=E_{\pi }\;\left[\sum_{k=0}^{\infty }\gamma^{k}r_{t+k}\right].$$

### 3.2. Options Formulation and Semi-Markov Decision Process

Temporally extended actions are modeled using the options framework [8], which extends the underlying MDP to a Semi-Markov Decision Process (SMDP) where each option may span multiple environment steps. An option $o_{i}$ is a tuple $\left(I_{i},\mu_{i},\beta_{i}\right)$ [9] with initiation set $I_{i}\subseteq S$, internal policy $\mu_{i}\left(a|s\right)$, and termination probability $\beta_{i}\left(s\right)$. The initiation set $I_{i}$ is the set of states from which $o_{i}$ can be invoked; $\mu_{i}$ selects low-level actions while the option is active; and $\beta_{i}\left(s\right)$ is the probability of terminating the option at state *s*. In this work, each subtask (Navigate, Pick, Place, Open, Close) is treated as an option, and the long-horizon mission is composed by chaining these options through the SMDP.

### 3.3. Skill Chaining

Long-horizon behavior is built by chaining options in sequence [9]: an option $o_{i}$ runs to termination, and the next option $o_{i+1}$ starts from the terminal state of $o_{i}$. For the chain to succeed, the terminal state of $o_{i}$ must lie within the initiation set $I_{i+1}$ of $o_{i+1}$. In standard skill chaining [9], $I_{i+1}$ is learned by an initiation set classifier over the continuous state space. In this work, we replace the learned classifier with an analytically defined region, described in Section 4.2, that gives a kinematically feasible hand-off between consecutive subtasks without requiring artificial resets.

### 3.4. Motion Planning Priors

Motion planning is concerned with finding a collision-free path from an initial configuration to a goal configuration within the configuration space C of a robot system [22]. The configuration space is partitioned into the obstacle-free region $C_{free}$ and the collision region $C_{obs}$, such that $C=C_{free}\cup C_{obs}$. A valid path is defined as a continuous mapping $\zeta :\left[0,1\right]\to C_{free}$ satisfying $\zeta \left(0\right)=q_{start}$ and $\zeta \left(1\right)=q_{goal}$, where $q_{start},q_{goal}\in C_{free}$ denote the initial and target configurations, respectively. Sampling-based planners address this problem by constructing a collision-free roadmap through incremental random sampling of $C_{free}$, avoiding the explicit construction of $C_{obs}$, which is intractable in high-dimensional spaces. The Rapidly-exploring Random Tree (RRT) [23] grows a tree T rooted at $q_{start}$ by iteratively sampling a random configuration $q_{rand}\in C$ and extending the nearest node $q_{near}\in T$ toward $q_{rand}$ by a step size η:

(2) $$q_{new}=q_{near}+\eta \;\frac{q_{rand}-q_{near}}{∥q_{rand}-q_{near}∥},\;\mathrm{if}\;\mathrm{CollisionFree}\left(q_{near},\;q_{new}\right).$$

Equation (2) is the standard RRT extension rule [23] and is not introduced by this work; its analytic properties, summarized below, are well established in the motion-planning literature. Each extension advances by a fixed step size, $∥q_{new}-q_{near}∥=\eta$, and a new node is added to the tree only when the connecting segment passes the collision and joint-limit check, so every accepted node lies in $C_{free}\subseteq C$. Since C is bounded by the manipulator’s joint limits, the generated configurations remain bounded and cannot diverge numerically. Probabilistic completeness is established in the standard RRT analysis [23], and the asymptotically optimal variant RRT* [24] converges to the optimal path through a local rewiring step that propagates cost improvements through the existing tree.

For mobile manipulator systems, the configuration space comprises both the base pose $q_{b}\in SE\left(2\right)$ and the articulated joint angles $q_{a}\in R^{n}$ (torso and arm), yielding a composite configuration $q=(q_{b},\;q_{a})\in C$ of dimension n+3. Planning directly in this high-dimensional joint space is computationally demanding, motivating a hierarchical decomposition of the planning problem [25]. In the hierarchical formulation, a global planner first computes a coarse waypoint sequence $\left\{\;q^{\left(0\right)},\;q^{\left(1\right)},\;\ldots ,\;q^{\left(K\right)}\;\right\}\subset C_{free}$ that connects $q_{start}$ to $q_{goal}$ at a task level, while a local planner subsequently resolves each inter-waypoint segment into a dynamically feasible trajectory subject to kinematic and collision constraints. This two-level structure reduces the effective planning horizon at each stage and has been shown to scale to the high-dimensional configuration spaces encountered in mobile manipulation [22,25].

Our MP-Augmented structure builds upon this hierarchical structure by employing a sampling-based planner to generate a reference trajectory $\zeta \in C_{free}$ in the full joint space of the mobile manipulator at the beginning of each training episode. Rather than using this trajectory solely as a kinematic plan to be tracked by a low-level controller, the computed path is integrated as a continuous geometric prior into the reinforcement learning reward function, providing dense per-step guidance throughout policy optimization.

## 4. MP-Augmented HRL for Long-Horizon Tasks

We present a hierarchical control architecture that combines motion planning with reinforcement learning. The long-horizon mission is split into a sequence of subtasks, each trained independently as a separate option [9]. Within each subtask, a collision-free reference trajectory from the motion planner is used as a per-step reward-shaping signal, guiding the RL agent through the joint configuration space. Between subtasks, a region-goal replaces the conventional point-to-point hand-off with a two-dimensional region of feasible base poses, removing the need for artificial teleportation between consecutive options.

**Figure 1:**
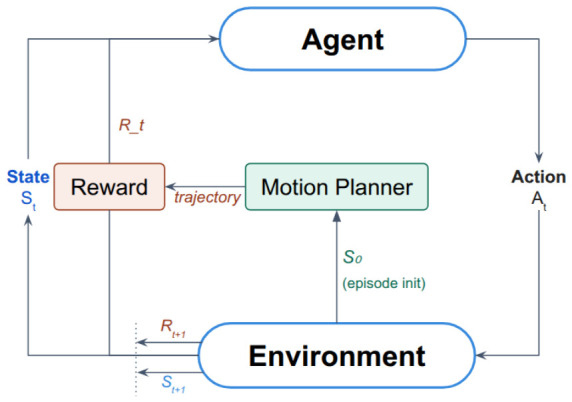
Overview of the MP-Augmented subtask training architecture. The motion planner (green) provides a reference trajectory that shapes the reward (orange), augmenting the standard agent–environment RL loop (blue).

Figure 1 illustrates how the motion planner is integrated into the RL training loop. At the beginning of each episode, the motion planner receives the initial state $s_{0}$ from the environment and computes a collision-free reference trajectory in the joint configuration space $C_{free}$. This trajectory is passed to the reward module, which combines it with the current state $s_{t}$ to compute the MP-guided tracking reward $R_{\mathrm{MP}}$ as part of the total reward $R_{\mathrm{total}}=R_{\mathrm{base}}+R_{\mathrm{col}}+R_{\mathrm{cond}}+R_{\mathrm{MP}}$, where $R_{\mathrm{base}}$ is the task-specific dense shaping term that encodes the subtask objective (such as TCP-to-object distance for Pick and object-to-goal distance for Place), $R_{\mathrm{col}}$ penalizes excessive contact forces against the environment, $R_{\mathrm{cond}}$ provides state-conditional shaping rewards based on task progress (pre-grasp, post-grasp, post-arrival, resting), and $R_{\mathrm{MP}}$ is the MP-guided tracking reward introduced in this work; complete mathematical formulations of these terms for each subtask are provided in Appendix A. The agent observes the augmented reward $R_{t}$ and selects action $A_{t}$, which the environment executes to produce the next state $s_{t+1}$ and reward $R_{t+1}$. Importantly, the motion planner is invoked only once per episode at initialization and does not intervene in the action selection loop; the agent retains full autonomy over its actions throughout the episode. Each subtask policy is trained independently via PPO or SAC in the HAB environment.

**Figure 2:**
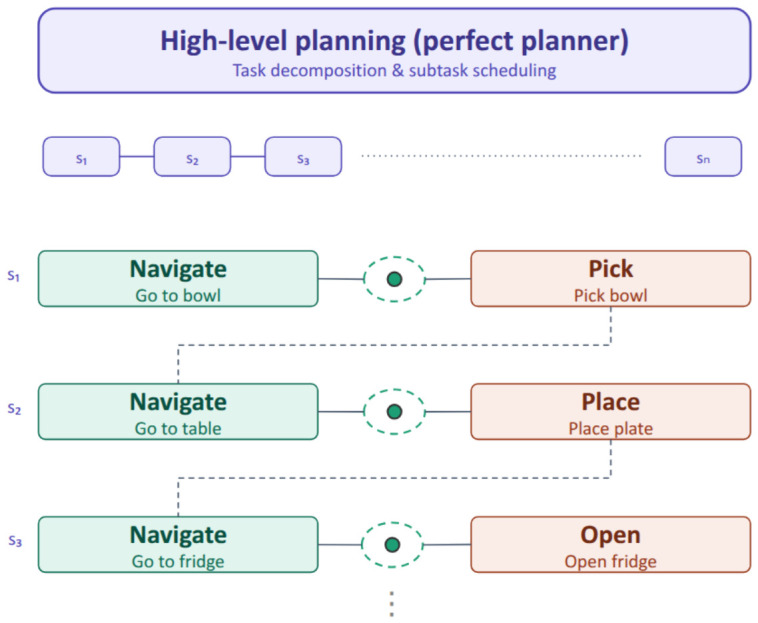
Overview of the SMDP-based skill-chaining pipeline for long-horizon task execution. Green and orange blocks denote navigation and manipulation subtasks, respectively; dashed circles mark transitions between consecutive skills.

Figure 2 illustrates the sequential evaluation pipeline. A perfect high-level planner decomposes the long-horizon mission into an ordered sequence of subtask pairs $\left(s_{1},s_{2},\ldots ,s_{n}\right)$, where each pair $s_{i}$ consists of a Navigate subtask followed by a manipulation subtask (Pick, Place, Open, or Close). The Navigate subtask targets a region-goal $G_{r}$ (shown as the dashed circle) around the subsequent manipulation target, ensuring that the robot arrives at a kinematically feasible pose before each manipulation phase begins. Each subtask inherits the exact terminal physical state of its predecessor as its initial state, eliminating the need for non-physical teleportation at test time.

### 4.1. HRL via SMDP-Based Skill Chaining

The Home Assistant Benchmark (HAB) [6] introduced apartment-scale long-horizon mobile manipulation as a standard evaluation framework for embodied AI, and has remained the reference benchmark for whole-body sequential rearrangement tasks since its release. The present work adopts ManiSkill-HAB (MS-HAB) [1], a recent GPU-accelerated reimplementation of HAB on the ManiSkill3 simulator that unifies four features in a single platform: simulation throughput exceeding 3× that of prior magical-grasp HAB implementations at a fraction of the GPU memory usage; full low-level contact-rich grasp control in place of the magical grasping used by the original HAB and by downstream methods such as M3 [20]; extensive RL and IL baselines for future work to compare against; and a rule-based trajectory filtering pipeline for controlled large-scale data generation. These features make MS-HAB a suitable public benchmark for evaluating the proposed architecture. The present work evaluates the proposed architecture on two long-horizon tasks drawn from this benchmark:

- TidyHouse: Five target objects are relocated from their initial positions to designated open receptacles such as tables and counters.
- SetTable: One bowl is retrieved from a closed drawer and one apple is retrieved from a closed fridge, both of which are subsequently placed on the dining table.

The long-horizon mission is modeled as a Semi-Markov Decision Process (SMDP) in which each subtask is treated as an option [8]. Five parameterized subtasks are defined as follows:

- Pick$\left(x_{pose}\right)$: grasps target object *x* given its ground-truth pose $x_{pose}=\left[x_{rot}|x_{pos}\right]$.
- Place$\left(x_{pose},\;g_{pos}\right)$: places object *x* at goal position $g_{pos}$, optionally within a target articulation.
- Open$\left(a_{pos}\right)$: opens articulation *a* (fridge or drawer) at handle position $a_{pos}$.
- Close$\left(a_{pos}\right)$: closes articulation *a* at handle position $a_{pos}$.
- Navigate$\left(G_{r}\right)$: repositions the mobile base to a region-goal $G_{r}$ defined around the subsequent manipulation target, rather than a single point coordinate.

Each subtask terminates with failure if the cumulative contact force applied by the robot exceeds a prescribed threshold. Successful execution of the long-horizon mission requires reliable hand-offs between consecutive options [2]. In practice, conventional approaches often fail at these hand-offs: the terminal state of the Navigate subtask does not always lie within the initiation set of the next manipulation subtask, causing the manipulation phase to fail on initialization [6]. Deriving an exact initiation set I that covers every feasible manipulation initialization is not tractable in complex indoor environments [9].

**Figure 3:**
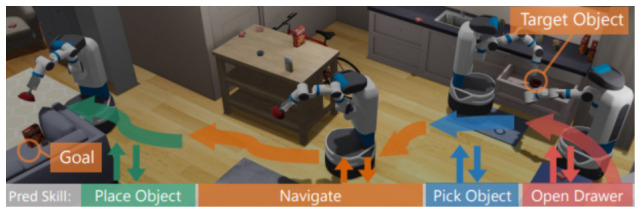
An example of long-horizon task skill chaining using region-goal navigation.

We address these hand-off failures with a region-goal navigation strategy. Following the region-goal formulation of M3 [20], a finite set of candidate base poses {g} is sampled around the target object; the union of poses from which the manipulation policy can succeed defines a two-dimensional region $G_{r}$. The Navigate option targets $G_{r}$ rather than a single point, so the terminal state of the Navigate subtask lies, by construction, within the initiation set of the next manipulation subtask. This removes the artificial teleportation step used in prior HAB-style protocols [6,20] and gives a kinematically feasible hand-off in continuous physical execution. Figure 3 illustrates an example of long-horizon skill chaining using region-goal navigation, adapted from [26].

### 4.2. Region-Goal Generation for Seamless Connectivity

Conventional navigation policies target a single fixed coordinate as the goal [2]. In the options framework, this point-to-point (P2P) formulation forces the termination of the navigation option onto a single point. If this point falls outside the initiation set I of the next option, the hand-off fails on initialization, and the failure rate compounds along the long-horizon chain.

M3 [20] relaxes this by replacing stationary manipulation with whole-body mobile manipulation. The feasible initial set $I_{\mathrm{manip}}$ is no longer a single configuration but a neighborhood of states from which the manipulation policy can succeed. The navigation policy is then trained with a region goal aligned with $I_{\mathrm{manip}}$, so small navigation errors no longer translate into manipulation failures.

The proposed system adopts the same idea, but defines the valid region analytically from the IK feasibility check in Appendix B.1. By deriving $G_{r}$ directly from IK constraints rather than from the empirical distribution of successful manipulation poses used in M3, we ensure by construction that every pose in the region is kinematically feasible for the subsequent subtask. The region goal $G_{r}$ is the set of collision-free base poses for which at least one IK solution exists:

(3) $$G_{r}=\left\{p\in C_{\mathrm{free}}|S\left(p\right)\neq \emptyset \right\},$$

where S(p) is the set of IK solutions for candidate base pose *p*. The resulting region extends up to approximately 2.0 m from the target object, reflecting the combined reach of the base and the arm. Any $p\in G_{r}$ satisfies $p\in I_{\mathrm{manip}}$ by construction, so the RL policy is free to choose any approach pose within $G_{r}$ that the training reward prefers.

### 4.3. MP-Augmented Subtask Learning

Prior work has used geometric priors as reward-shaping signals for whole-body control. Ota et al. [13] used per-step deviation penalties from RRT*-generated waypoints for a fixed-base 6-DoF manipulator, showing that planner-generated paths work as reward shaping signals without an explicit trajectory tracking module. Kindle et al. [7] trained an end-to-end RL policy for a mobile manipulator using a Harmonic Potential Field as a navigation reward. However, the Harmonic Potential Field is defined over a 2D plane, is prone to local minima in cluttered scenes, and does not capture the joint-space geometry needed for dexterous manipulation. Both works also target single-task settings and have no mechanism for kinematically feasible initialization in sequentially chained subtasks.

The proposed architecture replaces the analytic potential field with a sampling-based motion planner that operates directly in the full joint configuration space $C_{\mathrm{free}}$ of the mobile manipulator. Most prior reward-shaping works define reference paths in end-effector Cartesian space, where the planner gives a sequence of TCP positions as guidance targets. This is intuitive, but it ties the reward signal to inverse kinematics at every training step and does not capture whole-body coordination across the full kinematic chain. End-effector reference paths also become geometrically ambiguous near workspace boundaries because of IK multiplicity, especially when the robot is initialized far from the target. Generating reference waypoints directly in joint configuration space removes the IK ambiguity and gives a consistent guidance signal regardless of the initial base orientation. The joint-space planning cost stays manageable inside the training loop because the IK feasibility check is computed with a GPU-accelerated parallel solver, CuRobo [27].

At the start of each subtask episode, the planner computes a collision-free reference trajectory $\zeta :\left[0,1\right]\to C_{\mathrm{free}}$ from the current joint configuration $q_{\mathrm{start}}$ to the subtask goal configuration $q_{\mathrm{goal}}$. The trajectory is a sequence of waypoints $\left\{q^{\left(0\right)},q^{\left(1\right)},\ldots ,q^{\left(K\right)}\right\}\subset C_{\mathrm{free}}$ that is globally consistent and free of local minima. It is computed independently for each subtask policy defined in Section 4.1 and is used only as a reward shaping signal, not as a low-level commanded trajectory.

The MP-guided tracking reward $R_{\mathrm{MP}}$ is defined as:

(4) $$R_{\mathrm{MP}}=R_{\mathrm{IK}}+0.5\left(\frac{s_{\mathrm{traj}}}{L_{\mathrm{path}}}\right),$$

where the fractional term gives a dense progression bonus proportional to the current trajectory step $s_{\mathrm{traj}}$ relative to the total planned path length $L_{\mathrm{path}}$. The IK-based tracking reward $R_{\mathrm{IK}}$ penalizes the joint-space deviation from the planned waypoint:

(5) $$R_{\mathrm{IK}}=2\left(1-\tanh\;\left(2\;d_{t}^{\mathrm{joint}}\right)\right),$$

where $d_{t}^{\mathrm{joint}}={∥q_{t}-q_{t}^{*}∥}_{2}$ is the Euclidean distance between the current joint configuration $q_{t}$ and the target configuration $q_{t}^{*}$ on the planned trajectory (see Appendix B.1 for the IK target selection and Appendix A for the full reward formulations). Penalizing the full joint configuration, rather than only end-effector deviation, enforces whole-body coordination throughout the motion, which is needed for kinematically feasible terminal poses that fall within the initiation set of the next subtask. This shaping reduces undirected exploration in the early training phase and improves sample efficiency, especially when the target object lies outside the initial field of view. $R_{\mathrm{MP}}$ is added to the task-specific dense shaping terms; the per-subtask reward components are listed in Appendix A.

Algorithm 1 summarizes the MP-Augmented training procedure, with SAC shown as a representative example; PPO is used identically. The non-highlighted lines are the standard SAC loop, and the highlighted lines are the additions of this work: the per-episode motion-planner call and the per-step injection of the MP-shaped reward.
**Algorithm 1** MP-Augmented Soft Actor–Critic (SAC) algorithm.1:Initialize parameter vectors $\psi ,\bar{\psi },\theta ,\phi$2:**for** each iteration **do**3:      $\zeta \leftarrow M.\mathrm{Plan}(s_{0},G_{r})$4:      **for** each environment step **do**5:          $a_{t}∼\pi_{\phi }\left(a_{t}|s_{t}\right)$6:          $s_{t+1}∼P\left(s_{t+1}|s_{t},a_{t}\right)$7:          $r_{t}\leftarrow r\left(s_{t},a_{t}\right)+R_{\mathrm{MP}}\left(q_{t+1},\zeta \right)$8:          $D\leftarrow D\cup \{\left(s_{t},a_{t},r_{t},s_{t+1}\right)\}$9:      **for** each gradient step **do**10:        $\psi \leftarrow \psi -\lambda_{V}{\hat{\nabla }}_{\psi }J_{V}\left(\psi \right)$11:        $\theta_{i}\leftarrow \theta_{i}-\lambda_{Q}{\hat{\nabla }}_{\theta_{i}}J_{Q}\left(\theta_{i}\right)$ **for** i∈{1,2}12:        $\phi \leftarrow \phi -\lambda_{\pi }{\hat{\nabla }}_{\phi }J_{\pi }\left(\phi \right)$13:        $\bar{\psi }\leftarrow \tau \psi +\left(1-\tau \right)\bar{\psi }$

The motion planner is invoked exactly once at the beginning of each episode, conditioned on the initial state $s_{0}$ and the region-goal $G_{r}$ defined in Section 4.2; internally, M.Plan(·) comprises the IK target selection of Appendix B.1 followed by the hierarchical RRT* planning of Appendix B.2. The resulting trajectory ζ is held constant for the remainder of the episode. At every environment step, $R_{\mathrm{MP}}\left(q_{t+1},\zeta \right)$ evaluates the deviation of the current joint configuration from ζ and is added to the standard MS-HAB task reward $R_{\mathrm{task}}=R_{\mathrm{base}}+R_{\mathrm{col}}+R_{\mathrm{cond}}$ [1].

The motion planner does not participate in action generation: the agent retains full autonomy over $a_{t}$, and ζ enters the system exclusively through the reward. The RRT* planning cost is paid once per episode and amortized across all subsequent steps, while the per-step overhead reduces to a distance query against precomputed waypoints (Section 5.5).

### 4.4. Experimental Validation via Challenging Configurations

Under standard random initialization, the MS-HAB baseline policies already reach high subtask success rates [1], which leaves little room to see the effect of the MP-shaped reward. We therefore set up challenging scenarios on the ManiSkill-HAB benchmark that stress exploration and show why motion planning integration is needed. In these scenarios, the robot is spawned facing directly away from the target object during manipulation, so it must perform a full rotational search before it can see the target [1]. It is also initialized inside the narrow compartment of a fully opened door, which requires an evasive maneuver to escape. The baseline struggles with these geometric constraints and rarely finds a meaningful reward [4], whereas the motion planning prior gives our agent a direction-aware signal from the first step.

The evaluation is strictly teleport-free. We remove the artificial teleportation operators used in the baseline benchmark [2], and each subtask is initialized from the exact terminal physical state of the preceding one [1]. This setting tests how well the combined region-goal and MP-Augmentation system handles the errors that accumulate across sequential transitions under the same continuous-execution constraint a physical robot faces [3].

## 5. Results and Discussion

### 5.1. Experimental Setup and Ablation Conditions

We evaluate the proposed architecture in the ManiSkill-HAB simulation environment on the two long-horizon tasks defined in Section 4.1: TidyHouse (five target objects) and SetTable (one bowl and one apple). The number of core manipulation targets matches the MS-HAB [1] and M3 [20] benchmarks, ensuring fair comparability of task difficulty. However, the total subtask chain is longer in the present evaluation because the proposed framework performs physical navigation between every manipulation step in place of teleportation oracles. For TidyHouse, each of the five target objects requires a sequence of Pick, Navigate, and Place subtasks, yielding a chain of 19 subtasks once the initial Navigate and inter-object transitions are included. For SetTable, each of the two target objects requires a sequence of Open, Pick, Navigate, Place, Navigate, and Close subtasks, yielding a chain of 14 subtasks across the two cycles. The additional Navigate steps reflect the teleport-free execution protocol and further increase the difficulty of the long-horizon setting, so any numerical comparison against the MS-HAB baselines should be interpreted as a conservative estimate of the true performance advantage.

All experiments are conducted under challenging initialization, where every subtask spawns the robot at a standard random base position (x,y) as in MS-HAB [1], but with its orientation fixed to face exactly $180^{\circ }$ away from the target object, imposing a substantial exploration challenge in the complete absence of initial visual information. The evaluation is strictly teleport-free: each subtask inherits the terminal physical state of its predecessor as its initial state, eliminating the artificial teleportation oracles used in conventional benchmarks.

To show the contribution of the MP-shaped reward, we compare two ablation conditions that share an identical RL training pipeline and the same region-goal navigation subtask:

- Baseline (MSHAB subtasks + P2P NAV)—standard RL-trained subtask policies from the MS-HAB benchmark combined with point-to-point navigation. The manipulation subtasks are trained with the task-specific shaping terms defined in Appendix A but without the MP-guided tracking reward $R_{\mathrm{MP}}$.
- Ours (MPA subtasks + RG NAV)—MP-Augmented subtask policies trained with the MP-guided tracking reward $R_{\mathrm{MP}}$ defined in Equation (4), combined with region-goal navigation.

Both conditions use the same RL algorithms (PPO or SAC), the same network architectures, and the same region-goal navigation policy; the only difference is the presence of the MP-shaped reward term in the manipulation subtasks. The motion planner is never invoked at execution time in either condition and serves solely to supply the reward-shaping signal during training. The region-goal mechanism is adopted from M3 [20], where its effectiveness over point-goal navigation was established on the same HAB benchmark. Since the present ablation is designed to isolate the contribution of the MP-shaped reward rather than re-validate the region-goal formulation, both conditions share the same region-goal navigation policy as a controlled common component. We further note that a point-goal variant fails to complete the subtask chain under teleport-free evaluation in preliminary experiments, making fair long-horizon comparison infeasible. Since the proposed architecture integrates the MS-HAB subtask environment with the M3 region-goal navigation policy and adds MP-shaped reward shaping on top of this combined foundation, no single prior method occupies the same design point, precluding a direct one-to-one comparison. The ablation is therefore constructed to isolate the contribution of the MP-shaped reward by holding all other components, including the RL algorithm, network architecture, and region-goal navigation policy, constant across both conditions.

The observation and action spaces follow the standard MS-HAB protocol [1]. The observation consists of the robot’s joint configuration (base pose in SE(2), torso lift, 7-DoF arm joint angles), joint velocities, end-effector pose, gripper state, and the ground-truth pose of the target object or articulation. The action is a continuous control command specifying target joint velocities for the base, torso, and arm; the gripper open/close command is a separate binary action. Each subtask policy is trained independently via PPO [28] or SAC [29] using the default hyperparameters provided by the MS-HAB benchmark, including learning rate, batch size, discount factor, and network architecture. Training proceeds in an episodic manner (Algorithm 1): each episode begins with an environment reset, the agent samples actions from the policy at each environment step, the resulting transitions are stored in the replay buffer, and the policy parameters are updated at the end of each episode.

**Figure 4:**
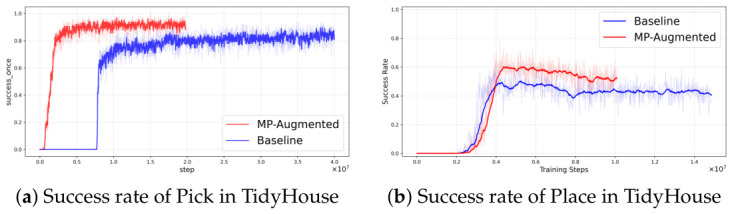
Learning curves for TidyHouse manipulation subtasks under challenging initialization.

**Figure 5:**
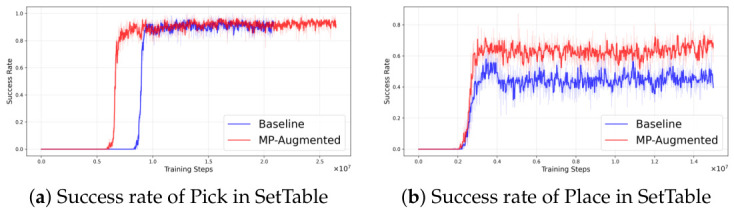
Learning curves for SetTable manipulation subtasks under challenging initialization.

**Figure 6:**
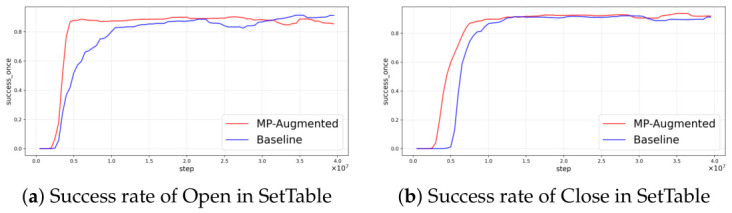
Learning curves for SetTable articulated manipulation subtasks (Open and Close) under challenging initialization.

All training hyperparameters, including learning rate, batch size, discount factor, network architecture, and the number of parallel environments, follow the default configurations provided by the MS-HAB benchmark [1]. All experiments were conducted on a single NVIDIA RTX 4090 GPU. Success rates are recorded at each training step across parallel environments, following the same benchmark’s logging protocol. The learning curves in Figure 4, Figure 5 and Figure 6 show exponentially smoothed success rates, and the shaded regions indicate the unsmoothed range. The final success rates reported in Table 2 correspond to the converged values at the end of training.

### 5.2. Subtask-Level Comparison

The robot must perform a rotational search to capture the target object when spawned facing away from the goal. Figure 4 and Figure 5 illustrate the learning curves for manipulation subtasks on the TidyHouse and SetTable tasks under these conditions. The Baseline tends to fail at obtaining meaningful rewards during the initial blind phase and experiences learning stagnation in the TidyHouse Pick and Place subtasks. Using the geometric trajectory guidance, our agent immediately rotates toward the target and initiates the approach. This geometric response allows the proposed agent to reach a high success rate well before 10 million training steps. The Baseline has difficulty overcoming the exploration challenge and maintains a low success rate in the challenging SetTable Place task. Ours provides a shorter path to the target coordinate through the MP-guided tracking reward $R_{\mathrm{MP}}$ and achieves stable convergence.

The tasks of opening and closing furniture require the robot to resolve complex kinematic constraints imposed by hinge structures rather than simply approaching an object. This manipulation difficulty is further compounded when the robot initializes facing completely away from the articulated furniture. In the challenging Open scenario, the Baseline fails to locate the handle and maintains a low success rate, whereas Ours directly guides the end-effector along the planned trajectory and achieves a high success rate. In the challenging Close scenario, the Baseline wanders aimlessly and fails to escape continuous collision penalties when trapped inside the narrow space of the open door. The collision-free geometric priors guide the agent through the evasive maneuver, keeping performance robust even under these conditions, as shown in Figure 6.

Table 2 summarizes the final subtask success rates and convergence steps under challenging initialization. Each success rate is averaged across all target objects within the corresponding task. Ours achieves competitive or superior success rates across all subtasks, with particularly pronounced gains in Place (+10.0 pp on TidyHouse) and Pick (+8.0 pp on SetTable), where early-stage exploration is most severely hindered by challenging initialization. Beyond final performance, the convergence speedup is most pronounced on SetTable Open, where Ours reaches its asymptotic success rate at approximately 5M training steps compared to 15M for the Baseline, a threefold reduction in training cost. Across all six subtasks, Ours converges at the same step as or earlier than the Baseline, indicating that the MP-shaped reward provides a consistent sample-efficiency benefit in addition to its final-performance advantage.

### 5.3. Region-Goal Navigation

**Figure 7:**
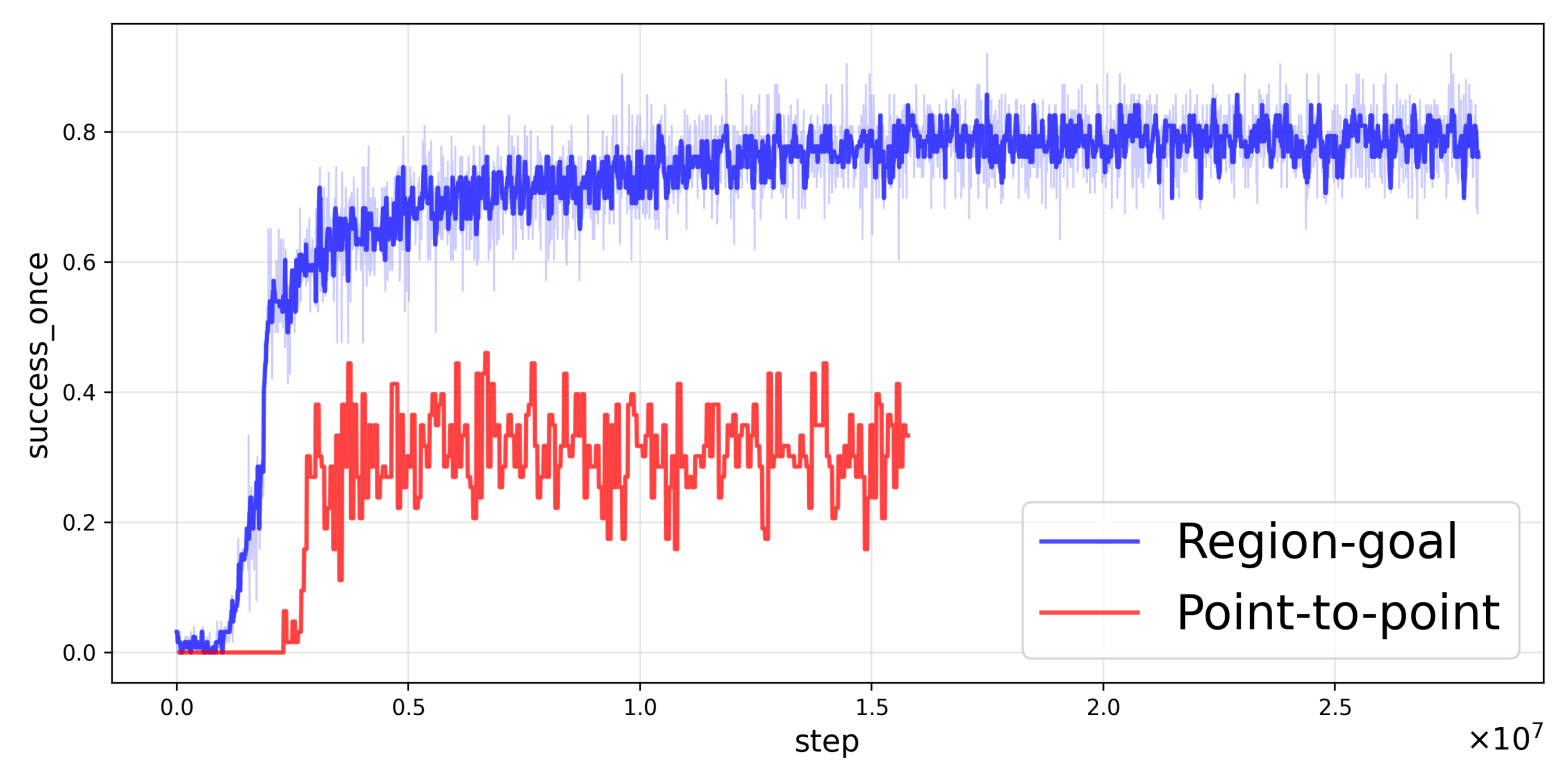
Success rate during training for the Navigate subtask.

Conventional baseline benchmarks sidestep the difficulty of mobile navigation by relying on artificial teleportation operators. This shortcut ignores the kinematic constraints and the errors that compound in real-world execution. To show that the teleportation oracle can be removed entirely, we evaluate a dedicated continuous navigation policy. Figure 7 shows the learning curves of the region-goal navigation policy trained with the full proposed pipeline (MP-Augmented RL with region-goal navigation) and of a minimal baseline configuration: standard RL with point-goal navigation, trained without $R_{\mathrm{MP}}$.

The point-goal baseline converges to approximately 30% success rate, whereas the full pipeline converges to approximately 76%, an improvement of about 46 percentage points. This reliable navigation performance bridges the physical hand-off gap between isolated manipulation subtasks, and the integrated region-goal mechanism allows the mobile base to consistently arrive at kinematically valid manifolds to maintain physical continuity throughout sequential execution. This comparison between the two boundary configurations of the design space demonstrates that replacing teleportation with a learned continuous navigation policy is feasible, and that the combination of region-goal formulation and MP-shaped reward is critical for achieving the navigation reliability required for teleport-free long-horizon execution.

### 5.4. Long-Horizon Sequential Performance

**Figure 8:**
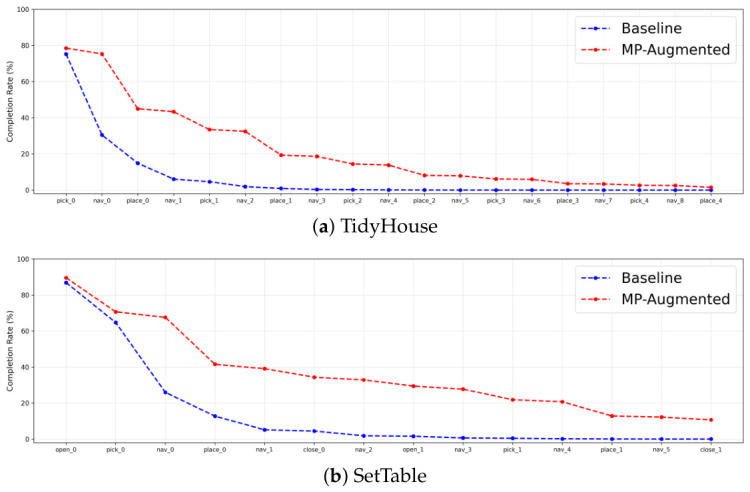
Sequential long-horizon evaluation under challenging initialization and teleport-free execution.

To evaluate end-to-end task performance, we execute the full subtask chain sequentially under the teleport-free protocol described in Section 5.1, where each subtask inherits the terminal physical state of its predecessor. The cumulative completion rate at each stage is obtained by progressively multiplying the per-subtask success rates, following the standard evaluation methodology of the MS-HAB [1] and M3 [20] benchmarks. The region-goal mechanism further supports this evaluation by ensuring that each manipulation subtask is initiated from within the kinematically feasible set, keeping the initial state distribution close to that seen during individual subtask training. Figure 8 reports the resulting cumulative completion rate along the full long-horizon chains, whose target counts match the MS-HAB and M3 benchmarks.

On the full TidyHouse chain of 19 subtasks (five object cycles, each a sequence of Pick, Navigate, and Place), Ours maintains a consistent margin over the Baseline throughout the entire sequence, with the gap between the two curves widening as each object is processed. The Baseline drops to near-zero after the first object cycle, whereas Ours sustains a measurable completion rate through all five cycles. The per-subtask advantage is most pronounced at the Place step (+10.0%), where early-stage exploration is most severely hindered by challenging initialization.

On the full SetTable chain of 14 subtasks (two complete cycles of Open, Pick, Place, and Close interleaved with the required Navigate transitions), the advantage of Ours grows with chain length: at the end of the first cycle Ours reaches ∼34% completion versus ∼5% for the Baseline, and maintains ∼10% at the end of the second cycle while the Baseline falls to near-zero, indicating that the benefit of MP-shaped reward compounds over the long-horizon sequence.

Although the absolute cumulative completion rate at the end of the SetTable chain (∼10%) is higher than that of TidyHouse (∼2%), this difference is primarily attributable to chain length rather than per-subtask difficulty: SetTable processes two target objects (14 subtasks) whereas TidyHouse processes five (19 subtasks), so the multiplicative attenuation over the longer chain drives the cumulative rate closer to zero. When comparing the per-cycle decay pattern, both tasks exhibit a similar compounding structure—the ratio between Ours and the Baseline widens at a comparable rate per object cycle, confirming that the MP-shaped reward provides a consistent per-subtask advantage that accumulates independently of the total chain length.

### 5.5. Discussion

The experimental results confirm that embedding collision-free joint-space trajectories as dense per-step reward signals effectively addresses the exploration bottleneck inherent in challenging initialization conditions. The core benefit of $R_{\mathrm{MP}}$ is its ability to provide immediate geometric feedback from the very first timestep of each episode, before the agent has any opportunity to observe the target object. In a standard RL setting, a robot spawned facing $180^{\circ }$ away from the goal receives no informative gradient signal until it completes a full rotational search, which in high-dimensional joint space can require thousands of random steps. The MP-guided tracking reward eliminates this blind phase by supplying a direction-aware shaping signal that rapidly orients the policy toward the target and allows Ours to enter the reward-rich manipulation regime well before 10 million training steps.

This benefit is most pronounced in subtasks that demand precise approach trajectories under tight spatial constraints, such as Pick from a closed fridge and Place on a cluttered counter, where the planned joint-space trajectory additionally provides whole-body coordination cues that suppress collisions during the approach. The disproportionate improvement on the Place subtask reflects the compound difficulty unique to placement. Unlike Pick, which terminates upon a successful grasp, Place requires the policy to simultaneously stabilize the grasped object during transport, align both the object and the TCP with the goal position, regulate the placement height, and retract the arm after release (Appendix A.2). The MS-HAB benchmark reflects this asymmetry by assigning a higher cumulative collision-force threshold to Place (7500 N) than to Pick (5000 N) and by classifying Place as the most difficult subtask among the four core skills [1]. Under challenging configurations, this difficulty is amplified because the robot must execute a whole-body reorientation while holding the object, which increases collision risk relative to the empty-handed rotation required in Pick. $R_{\mathrm{MP}}$ directly addresses this geometric component by supplying a collision-free whole-body path from the first timestep, complementing the existing task-specific terms $R_{\mathrm{base}}$ and $R_{\mathrm{cond}}$. Supplementing the remaining reward terms with analogous dense guidance signals for the transport and post-release phases could further close the performance gap between Place and the other subtasks. Subtasks with relatively open workspaces, such as Close, show smaller gains, as the Baseline can discover feasible trajectories through less directed exploration once the target enters the field of view. Even modest per-subtask improvements compound into substantial gains at the task level, as evidenced by the widening gap between the two curves in Figure 8. The region-goal navigation policy contributes to this effect by achieving a stable asymptotic success rate of approximately 76% within 2.5 million environment steps, demonstrating that a continuous locomotion policy can fully replace the teleportation oracle used in prior benchmarks. The spatial manifold provides the RL policy with sufficient flexibility to discover kinematically optimal approach configurations, and the arrival condition $p\in G_{r}$ structurally satisfies the initiation set of the subsequent manipulation option, eliminating compounding hand-off failures without any artificial reset.

A direct numerical comparison with the MS-HAB baselines requires careful interpretation due to the fundamental asymmetry in evaluation conditions. The MS-HAB RL-Per policy is trained and evaluated under standard random initialization with kinematically favorable spawn poses and relies on a teleportation oracle to transition between subtasks. Ours, by contrast, faces challenging initialization and must navigate physically from one subtask to the next without any artificial reset. Despite this substantially harder evaluation protocol, Ours achieves comparable or superior subtask success rates in the majority of evaluated subtask types, as shown in Table 2.

The MS-HAB baseline [1] is initialized facing the target, where the kinematically favorable spawn poses let it discover feasible trajectories through undirected exploration within a reasonable number of training steps; in this setting, the gain from $R_{\mathrm{MP}}$ is small. Given that the per-episode planning overhead of RRT* is confined to episode initialization and the per-step reward computation reduces to a distance query against precomputed waypoints, the cost–benefit trade-off in that setting remains modest. The challenging initialization protocol is therefore designed to isolate the regime where geometric guidance becomes essential: when the robot has no initial visual contact with the target and must execute a coordinated whole-body reorientation before any task-relevant reward can be obtained. In this regime, the MP-shaped reward transforms an otherwise intractable exploration problem into a structured guided search; the planning overhead is amortized by the acceleration in convergence and improved sample efficiency (Table 2).

To quantify the computational cost of the proposed MP-shaped reward, we measured the training throughput on the SetTable Open subtask under matched configurations on a single NVIDIA RTX 4090 GPU. The throughput of the proposed method (with $R_{\mathrm{MP}}$) is essentially identical to that of the Baseline (without $R_{\mathrm{MP}}$), indicating that the additional planning and reward-shaping pipeline introduces negligible wall-clock overhead, within measurement noise. This near-zero overhead arises from two structural properties of the proposed integration. First, the motion planner is invoked exactly once per episode at initialization (Algorithm 1), so the RRT* planning cost is amortized across all subsequent environment steps within that episode; the per-step reward computation $R_{\mathrm{MP}}\left(q_{t+1},\zeta \right)$ reduces to a distance query against precomputed waypoints whose cost is dominated by the GPU-parallel simulation throughput of MS-HAB. Second, the IK feasibility check that constructs the region-goal $G_{r}$ and selects the planning target is computed via CuRobo version 0.6.2 [27], a GPU-accelerated parallel IK solver that processes the candidate poses concurrently, keeping the per-episode planning step well within the cost budget absorbed by the surrounding simulation loop.

The current evaluation is conducted entirely within the ManiSkill-HAB simulation environment, and we acknowledge that physical deployment introduces sim-to-real challenges including sensor noise, unmodeled friction, and contact dynamics mismatch. Physical-robot validation is planned as the immediate next step but was not pursued in the present work due to time and equipment constraints; we instead focused on establishing the algorithmic foundation on a rigorous public simulation benchmark. Within these constraints, the proposed architecture incorporates three concrete provisions that close substantive portions of the sim-to-real gap relative to the standard HAB-style protocol. First, our evaluation eliminates the artificial teleportation operators commonly used in prior HAB-style benchmarks [6,20], requiring each subtask to inherit the exact terminal physical state of its predecessor; this protocol enforces the same continuous-execution constraint that a physical robot must satisfy, in which the base and arm cannot be repositioned by an external oracle between subtasks. Second, the region-goal delineates the operational region of the subsequent manipulation subtask analytically from the manipulator’s IK feasibility (Equation (3)), rather than from heuristic spatial templates; every hand-off learned in simulation is therefore, by construction, a base configuration from which the subsequent manipulation subtask is kinematically executable on the same robot model in the physical world. Third, unlike prior HAB-based evaluations that employ magical grasping, our setting requires the policy to execute full low-level grasp control [1], so the learned policies acquire genuine contact-rich grasping behaviors that constitute the single largest source of sim-to-real failure in mobile manipulation.

Remaining gaps to physical deployment include sensor noise, unmodeled actuation dynamics, and vision-based perception. Sensor noise enters the policy through the base-pose observation, and we plan to address it at two points along our pipeline: training the low-level policy on domain-randomized base-pose observations so that it adapts to the estimation-noise distribution at inference rather than relying on noise-free state [30], and using a learned degradation signal that triggers closed-loop replanning of the reference trajectory ζ when accumulated estimation error grows, in line with degradation-aware sensor-compensation strategies developed for related robotic systems [31]. Unmodeled actuation effects such as joint friction will be mitigated at deployment by replacing the idealized simulator actuator with an actuator network calibrated on hardware measurements, complemented by a teacher–student scheme that recovers privileged dynamics information from proprioceptive history; analogous stability-control strategies have been demonstrated on bio-inspired robotic platforms [32]. Vision-based pose-estimation bias will be absorbed by a residual correction module operating on the discrepancy between visual and proprioceptive state estimates. Integrating these adaptive correction modules with a GPU-accelerated replanning back-end such as CuRobo [27] is the natural next step toward physical deployment.

Finally, the task planner used in this work is a perfect oracle that supplies the subtask sequence directly to our hierarchical controller. Replacing this oracle with a task-level planner that autonomously generates subtask sequences from language instructions or perceptual inputs, such as a Large Language Model coupled with Vision-Language Model verification, would substantially broaden the applicability of the proposed method to open-ended household manipulation. Such integration is orthogonal to the contributions evaluated here, since the low-level region-goal hand-off mechanism does not depend on how the subtask sequence is produced.

## 6. Conclusions

This work addressed three fundamental challenges in long-horizon mobile manipulation identified in the introduction: reward sparsity, exploration inefficiency, and sequential connectivity failure. The hierarchical SMDP-based decomposition resolves reward sparsity by partitioning the overarching mission into independently learnable subtasks, each with a well-defined termination condition that provides dense intermediate feedback. The MP-Augmented reward shaping directly targets exploration inefficiency by embedding collision-free joint-space trajectories generated by OMPL/RRT* as per-step guidance signals, eliminating the blind exploration phase that standard RL agents face under challenging initialization. The region-goal mechanism addresses the hand-off problem by replacing rigid point-to-point subtask hand-offs with a continuous spatial manifold, ensuring that the terminal state of each navigation phase lies within the kinematically feasible initiation set of the subsequent manipulation policy without requiring non-physical teleportation. Experimental results on the ManiSkill-HAB benchmark confirm that these three contributions jointly achieve improvements in sample efficiency and execution robustness under challenging initialization conditions, with the performance advantage compounding across the task chain as the number of sequential subtasks increases.

Looking further ahead, the present work relies on a perfect task planner that provides a fixed subtask sequence. Replacing this oracle with Large Language Model- and Vision-Language Model-based task-level reasoning would allow the system to parse open-ended human instructions, verify intermediate progress from visual observations, and dynamically compose subtask chains for previously unseen household missions. A complementary direction is to fully integrate the option-theoretic formulation of the SMDP framework to support subtask interruption, so that the currently executing option can be terminated and replaced in response to unexpected state changes, execution failures, or updated task specifications rather than running to completion in a strictly sequential manner. Together, these extensions would move the architecture toward a fully autonomous agent capable of reactive long-horizon manipulation in unstructured real-world environments.

## Figures and Tables

**Table 1:** Comparison with prior RL-based works.

Method	Simulator	Task	MP Integ.	Ref. Space	SC	RG	HRL	Grasp
PRM-RL [5]	Custom 2D	2D Nav.	Sub-goal	Cartesian	×	×	×	—
Kindle et al. [7]	Gazebo	WB Reaching	Reward	Cartesian	×	×	×	—
MoPA-RL [4]	MuJoCo	Manipulation	Action-space	Joint	×	×	×	—
Ota et al. [13]	PyBullet	Reaching	Reward	Joint	×	×	×	—
ReLMoGen [2]	iGibson	Interactive Nav	—	—	√	×	√	Magical
M3 [20]	Habitat 2.0	HAB	—	—	√	√	√	Magical
PSL [11]	Robosuite	Manipulation	Action-space	Cartesian	√	×	√	Magical
PLANRL [10]	RoboSuite	Manipulation	Mode-switch	Mixed	×	×	×	—
ARCH [3]	IsaacGym	Manipulation	Hybrid	Cartesian	√	×	√	—
MS-HAB [1]	ManiSkill3	HAB	—	—	√	×	√	Realistic
Ours	ManiSkill3	HAB	Reward	Joint	√	√	√	Realistic

Abbreviations: MP Integ. = motion planning integration modality; Ref. Space = reference space of the planner output; SC = skill chaining with sequential navigation between subtasks; RG = region-goal navigation; HRL = hierarchical reinforcement learning; WB = whole body. √ indicates the feature is supported; × indicates it is not; (—) indicates not applicable.

**Table 2:** Subtask-level ablation under challenging initialization.

Task	Subtask	Ours (MP-Aug)		Baseline	
		SR (%)	Conv. (M)	SR (%)	Conv. (M)
TidyHouse	Pick	78.0	∼8	75.0	∼10
	Place	60.0	∼4	50.0	∼4
SetTable	Open	89.0	∼5	87.0	∼15
	Pick	91.0	∼8	83.0	∼10
	Place	68.0	∼3.5	65.0	∼4
	Close	89.0	∼7	88.0	∼10

## Data Availability

This study was conducted using the publicly available ManiSkill-HAB benchmark (https://github.com/arth-shukla/mshab (accessed on 10 June 2026)).

## Abbreviations

The following abbreviations are used in this manuscript:

MP	Motion Planning
RL	Reinforcement Learning
DRL	Deep Reinforcement Learning
MSHAB	ManiSkill-HAB
HAB	Home Assistant Benchmark
TCP	Tool Center Point
EE	End Effector
Nav	Navigate
SAC	Soft Actor–Critic
PPO	Proximal Policy Optimization
SMDP	Semi-Markov Decision Process
MDP	Markov Decision Process
HRL	Hierarchical Reinforcement Learning
LLM	Large Language Model
VLM	Vision-Language Model
IK	Inverse Kinematics
P2P	Point-to-point

## Appendix A. Detailed Subtask Reward Functions

Our MP-Augmented architecture defines specific reward functions tailored to the distinct kinematic constraints of each subtask. As defined in Section 4, all manipulation and navigation subtasks share the unified structural formulation $R_{total}=R_{base}+R_{col}+R_{cond}+R_{MP}$. This appendix exhaustively details the specific mathematical formulations of the non-MP terms for Pick, Place, Open, Close, and Navigate.

### Appendix A.1. Pick Subtask

The base reward $R_{base}$ for the Pick subtask encourages the Tool Center Point (TCP) to reach the object, stabilizes the end-effector velocity ($v_{ee}$), and provides sparse bonuses for grasping and ultimate task success. The mathematical definition is given by:

(A1) $$R_{base}=5\left(1-\tanh\left(3d_{to}\right)\right)+\left(1-\tanh\left(v_{ee}/5\right)\right)+2\cdot I_{grasped}+3\cdot I_{success}$$

Here, $d_{to}=∥p_{tcp}-p_{obj}∥$ represents the Euclidean distance to the target object. The collision penalty $R_{col}$ penalizes instantaneous interaction forces *f* that exceed a safety threshold $f_{min}$, and limits the cumulative force $F_{cum}$ during the delicate approach.

(A2) $$R_{col}=3\left(1-\tanh\left(3\left(\max\left(\alpha f,f_{min}\right)-f_{min}\right)\right)\right)+2\cdot I\left(F_{cum}<F_{limit}\right)$$

The state-conditional guidance $R_{cond}$ applies distinct shaping rewards based on the task progress, separating the pre-grasp, post-grasp, and resting phases.

(A3) $$R_{cond}=\left(1-I_{grasped}\right)\cdot r_{pre}+I_{grasped}\cdot r_{post}+I_{rest}\cdot r_{stable}$$

We explicitly define the individual phase rewards to stabilize the torso before grasping and retract the arm safely once the object is secured.

(A4) $$r_{pre}=\left(1-\tanh(5\right|{\dot{q}}_{torso,z}|))+(1-\tanh(5∥p_{obj,xy}-p_{tcp,xy}∥))$$

(A5) $$r_{post}=2+5\left(1-\tanh\left(3d_{rest}\right)\right)+\left(1-\tanh\left(v_{base}\right)\right)+r_{arm\_rest}$$

(A6) $$r_{stable}=2+(1-\tanh(∥q_{vel}^{all}∥))$$

Here, $d_{rest}$ represents the deviation from the resting pose, and $v_{base}$ is the mobile base velocity.

### Appendix A.2. Place Subtask

The Place subtask shifts the base reward focus toward stabilizing the object velocity ($v_{obj}$) during transportation and securing the target placement goal. The penalty for object velocity is strictly deactivated during the active grasp phase.

(A7) $$R_{base}=\left(1-\tanh\left(v_{ee}/5\right)\right)+3\cdot \left(1-I_{grasped}\right)\left(1-\tanh\left(v_{obj}/5\right)\right)+6\cdot I_{success}$$

Our method utilizes the identical collision penalty $R_{col}$ as defined in the Pick subtask to strictly avoid destructive forces against the environment or placement surface. The state-conditional guidance $R_{cond}$ provides phased shaping, depending on whether the object is being transported, has reached the goal, or the robot is returning to a resting pose.

(A8) $$R_{cond}=I_{not\_goal}\cdot r_{trans}+I_{at\_goal}\cdot r_{post}+I_{rest}\cdot r_{stable}$$

We formulate the specific phase rewards to minimize the object-to-goal distance ($d_{og}$) and the TCP-to-goal distance ($d_{tg}$), while strictly regulating the placement height ($r_{height}$).

(A9) $$r_{trans}=2\cdot I_{grasped}+(1-\tanh(5|{\dot{q}}_{torso,z}\left|)\right)+6\left(\frac{1-\tanh\left(d_{og}\right)+\tanh\left(d_{tg}\right)}{2}\right)+r_{height}$$

(A10) $$r_{post}=13+5\left(1-\tanh\left(3d_{rest}\right)\right)+4(1-\tanh(∥q_{arm}-q_{rest}∥))+2\cdot r_{torso\_rest}+\left(1-\tanh\left(v_{base}\right)\right)$$

(A11) $$r_{stable}=2+(1-\tanh(∥q_{vel}^{all}∥))+\left(1-\tanh\left(v_{base}\right)\right)$$

### Appendix A.3. Open Subtask

Opening an articulated object demands continuous contact and precise force control throughout the interaction. The total reward $R_{open}$ sequentially combines reaching, articulation progress, slow-motion regularization, and post-interaction resting.

(A12) $$R_{open}=r_{reach}+r_{open}+r_{slow}+r_{rest}+r_{MP}+r_{success}$$

The reaching term $r_{reach}$ and the articulation progress term $r_{open}$ guide the end-effector to the handle ($d_{tcp}$) and push the joint past the required threshold ($f_{open\_left}$).

(A13) $$r_{reach}=3\left(1-\tanh\left(\alpha \cdot d_{tcp}\right)\right)+5\cdot I_{grasped}+6\cdot I_{is\_open}$$

(A14) $$r_{open}=6\left(1-f_{open\_left}\right)+3\cdot I\left(q_{pos}>q_{min}+0.01\right)$$

We define a slow-motion reward $r_{slow}$ that encourages maintaining an ideal joint velocity $v_{ideal}$ to prevent excessive collision forces during the dynamic articulation.

(A15) $$r_{slow}=3\cdot I(|q_{vel}\left|\leq v_{ideal}\right)$$

The resting reward $r_{rest}$ forces the robot to return to a neutral posture, but it actively triggers only after the articulation is successfully opened.

(A16) $$r_{rest}=I_{is\_open}\cdot \left(r_{arm\_torso}+r_{torso\_pos}+r_{ee\_dist}+r_{static}\right)$$

### Appendix A.4. Close Subtask

The Close subtask utilizes a similarly shaped reward structure, but we modify the weights to prioritize maintaining contact while pushing the articulation to a fully closed state.

(A17) $$R_{close}=r_{reach}+r_{close}+r_{slow}+r_{rest}+r_{MP}+r_{success}$$

Our formulation updates the reaching term and the closing progress term to strongly enforce proximity to the handle during the final closing phase.

(A18) $$r_{reach}=7\left(1-\tanh\left(\alpha \cdot d_{tcp}\right)\right)+7.5\cdot I_{grasped}+8\cdot I_{closed\_near}$$

(A19) $$r_{close}=5\left(1-f_{close\_left}\right)$$

The slow-motion reward $r_{slow}$ mirrors the Open task but applies a higher penalty weight for velocity violations to ensure safe physical interactions.

(A20) $$r_{slow}=5\cdot I(|q_{vel}\left|\leq v_{ideal}\right)$$

The stabilization term $r_{rest}$ amplifies the resting penalty for the arm and torso by a factor of 10 upon successful closure to guarantee structural safety and prevent subsequent kinematic entrapment.

(A21) $$r_{rest}=I_{closed\_near}\cdot \left(10\cdot r_{arm\_torso}+r_{torso\_pos}+r_{ee\_dist}+r_{static}\right)$$

### Appendix A.5. Navigate Subtask

The primary objective of the Navigate subtask is to safely and efficiently move the robot base into the valid RG $G_{r}$ established by our methodology. The base reward $R_{base}$ reduces the relative distance to the goal, penalizes elapsed time $\lambda_{slack}$, and stabilizes the arm posture during transit.

(A22) $$R_{base}=\lambda_{dist}\left(1-\tanh\left(\frac{\Delta d}{scale}\right)\right)+4\cdot I_{grasped}+\lambda_{succ}\cdot I_{at\_goal}+r_{ee\_still}+r_{arm\_rest}-\lambda_{slack}$$

Our architecture identically applies the collision penalty $R_{col}$ from the manipulation phases to ensure strict obstacle avoidance throughout the mobile navigation phase. The conditional term $R_{cond}$ enforces base deceleration and kinematic recovery strictly after the robot successfully reaches the target region.

(A23) $$R_{cond}=I_{at\_goal}\cdot r_{post}+I_{rest}\cdot r_{stable}$$

We define the post-arrival recovery terms to heavily incentivize stopping the mobile base and retracting the arm, structurally preventing hand-off failures before the next subtask initiates.

(A24) $$r_{post}=3+5\left(1-\tanh\left(3d_{rest}\right)\right)+r_{arm\_rest}+\left(1-\tanh\left(v_{base}\right)\right)+r_{ee\_still}$$

(A25) $$r_{stable}=2+(1-\tanh(∥q_{vel}^{all}∥))$$

## Appendix B. Detailed Motion Planning

### Appendix B.1. Inverse Kinematics Target Selection

Optimal kinematic targets for each manipulation subtask are computed using the CuRobo library [27]. Candidate mobile base poses are generated by uniformly sampling positions on two concentric rings surrounding the target object at radial distances r∈{0.45,0.55} m, each divided into N=8 discrete angular segments, yielding up to 16 candidates per subtask.

Each candidate base pose $p_{i}\in SE\left(2\right)$ is transformed into the local coordinate frame of that pose, and a batched inverse kinematics solver is executed across all candidates simultaneously. Successful IK solutions are scored by

(A26) $$s_{i}=d_{i}+w\;{∥\Delta q_{i}∥}_{2},$$

where $d_{i}$ is the Euclidean distance from the current base position to candidate $p_{i}$, $\Delta q_{i}$ is the deviation of the solved arm configuration from the home configuration, and w>0 is a scalar weight that balances base travel against joint articulation. The final target is the candidate achieving the lowest score:

(A27) $$i^{*}=\arg\min_{i\in S}\;s_{i},$$

where S denotes the set of candidates for which IK succeeded.

### Appendix B.2. Hierarchical Motion Planning

Hierarchical motion planning is a widely adopted strategy for mobile manipulators that decomposes the high-dimensional planning problem by treating the mobile base and the manipulator arm as separate subsystems [33,34]. Given the selected target $\left(p^{*},q^{*}\right)$ from Equation (A27), planning proceeds in two stages.

Stage 1 (Base navigation). A collision-free base path $\pi_{\mathrm{base}}:\left[0,1\right]\to C_{\mathrm{base}}$ is computed in the 2D configuration space $C_{\mathrm{base}}=SE\left(2\right)$ from the current base pose to $p^{*}$ using RRT* via OMPL [35].

Stage 2 (Arm articulation). With the base path fixed, a collision-free arm trajectory $\zeta_{\mathrm{arm}}:\left[0,1\right]\to C_{\mathrm{arm}}$ is planned in the joint configuration space $C_{\mathrm{arm}}\subset R^{n}$ from the current arm configuration $q_{0}$ to the target $q^{*}$, subject to collision constraints evaluated along $\pi_{\mathrm{base}}$.

The combined trajectory $\zeta =(\pi_{\mathrm{base}},\;\zeta_{\mathrm{arm}})$ is guaranteed to be collision-free throughout execution. Per-step deviations from ζ are subsequently incorporated into the reward function as dense geometric shaping signals, guiding the RL policy toward kinematically feasible and collision-free behavior [13].
